# Intestinal microecology: a crucial factor influencing incomplete immune reconstitution after antiretroviral therapy in people living with HIV-1

**DOI:** 10.3389/fpubh.2026.1729968

**Published:** 2026-02-02

**Authors:** Li Chen, Zhang Xinxin, Zhang Yue, Xu Qianlei, Guo Huijun, Liu Xuewei

**Affiliations:** 1 Department of AIDS Clinical Research Center, The First Affiliated Hospital of Henan University of Chinese Medicine, Zhengzhou, China; 2 Department of Dermatology, The First Affiliated Hospital of Henan University of Chinese Medicine, Zhengzhou, China

**Keywords:** HIV-1 infection, immune reconstitution, incomplete immune reconstitution, intestinal microecology, therapeutic interventions

## Abstract

Some people living with HIV-1 (PLWH) experience insufficient increases in CD4 + T cell counts after antiretroviral therapy (ART), a clinical manifestation referred to as incomplete immune reconstitution (INR). INR significantly increases in the incidence of AIDS and non-AIDS events and profoundly affects the life expectancy and quality of life of PLWH. Recent studies have indicated that intestinal microecology plays a crucial role in immune reconstitution through multiple pathways. This review summarizes several mechanisms through which intestinal microecology contributes to impaired immune reconstitution in PLWH, including changes in microbiota composition, variations in intestinal metabolic products, and damage to the intestinal mucosal barrier. Additionally, intervention strategies such as fecal microbiota transplantation, probiotics, and traditional Chinese medicine are proposed. These innovative therapeutic approaches hold promise for overcoming the limitations of conventional treatments, providing clinicians with a scientific basis for personalized therapeutic strategies and researchers with theoretical guidance for exploring novel mechanisms and research methods. Ultimately, these efforts aim to improve the prognosis and quality of life for PLWH and reduce the global public health burden posed by HIV-1 infection.

## 1 Introduction

Antiretroviral therapy (ART) is the primary treatment for AIDS, effectively suppressing HIV-1 replication, increasing CD4 + T cell counts, and promoting immune reconstitution in PLWH (1). However, approximately 10–40% of PLWH do not achieve sufficient elevation or restoration of CD4 + T cell counts despite continuous ART. These patients experiencing INR following ART are termed immunological non-responders (INRs) (2, 3). Currently, no consensus exists regarding a precise definition of INR. Research and guidelines typically define INR based on criteria such as CD4 + T cell counts below baseline, elevated relative to baseline, or a percentage increase relative to baseline (4). The World Health Organization (WHO) defines INR as a “CD4+ T cell count <250/μL or persistently <100/μL after 6 months of effective treatment” (5). In contrast, the US Department of Health and Human Services (DHHS) defines INR as “patients with a CD4+ T cell count increase of 50–150/μL within the first year of ART treatment.” A systematic review synthesizing 73 guideline criteria for INR identified the most common definition as “sustained viral suppression for over 2 years with a CD4+ T cell count <350/μL” (6). Compared to CD4 + T cell counts and viral load, the CD4+/CD8 + ratio also provides significant predictive and evaluative value regarding immune recovery in patients (7).

Persistently low CD4 + T cell counts in these patients significantly increase the risk of AIDS and non-AIDS events, such as malignancies, metabolic syndrome, cardiovascular diseases, and liver and kidney disorders. Furthermore, INR accelerates AIDS progression and has become a significant risk factor for increased mortality among PLWH (8, 9). The development of INR is likely influenced by multiple incompletely understood factors. Together with the insidious onset of early clinical symptoms, this limited understanding of targeted interventions adversely impacts the quality of life for PLWH. The precise mechanisms underlying INR remain highly challenging (10). Recent research indicates that intestinal microbiota plays a critical role in the development of INR, with specific intestinal microbiota characteristics potentially providing insights into its underlying mechanisms (11–13). Therefore, this review examines the relationship between intestinal microecology and INR pathogenesis from the perspective of gut microbiota, aiming to offer new directions for future INR-related research and therapeutic solutions.

## 2 The human intestinal microecology

Human intestinal microecology is a complex microbial community consisting of trillions of microorganisms inhabiting the nutrient-rich intestinal tract and represents an integral component of the human body’s internal environment (14). The intestinal microbiota includes more than 1,000 microbial species, such as bacteria, fungi, viruses, and other microorganisms. Specific phyla, including *Firmicutes*, *Bacteroidetes*, *Actinobacteria*, and *Proteobacteria*, predominate (15, 16). The total number of genes encoded by these microorganisms exceeds those in the human genome by approximately 100-fold (17). The intestinal microbiome constitutes the most important microecosystem in the human body, functioning as a “hidden organ” that coexists symbiotically with its host (18). The intestinal microbiome not only influences intestinal health but also helps regulate the host’s immune system, material and energy metabolism, and neuromodulation, thus promoting the development and maturation of the immune system. Additionally, the intestinal microbiome contributes to constructing the intestinal mucosal barrier, effectively preventing pathogenic microbes from colonizing and proliferating within the intestinal tract (19). Therefore, maintaining the dynamic balance of intestinal microbiota is essential for human health. Under normal circumstances, intestinal microecology maintains a complex equilibrium with the host. Once disrupted, this balance becomes closely associated with various human diseases, including cardiovascular, gastrointestinal, neurological, and metabolic disorders. Consequently, intestinal microbiome-oriented studies serve as bridges to reveal underlying disease mechanisms (20–22). In recent years, high-throughput sequencing technology has been widely applied in microbiology research, rapidly transitioning from basic research to clinical practice (23). In AIDS research, studies exploring intestinal microbiota-associated pathogenesis have emerged as promising avenues to address therapeutic challenges in this field.

## 3 Correlation between intestinal microecology and INR

**Figure 1:**
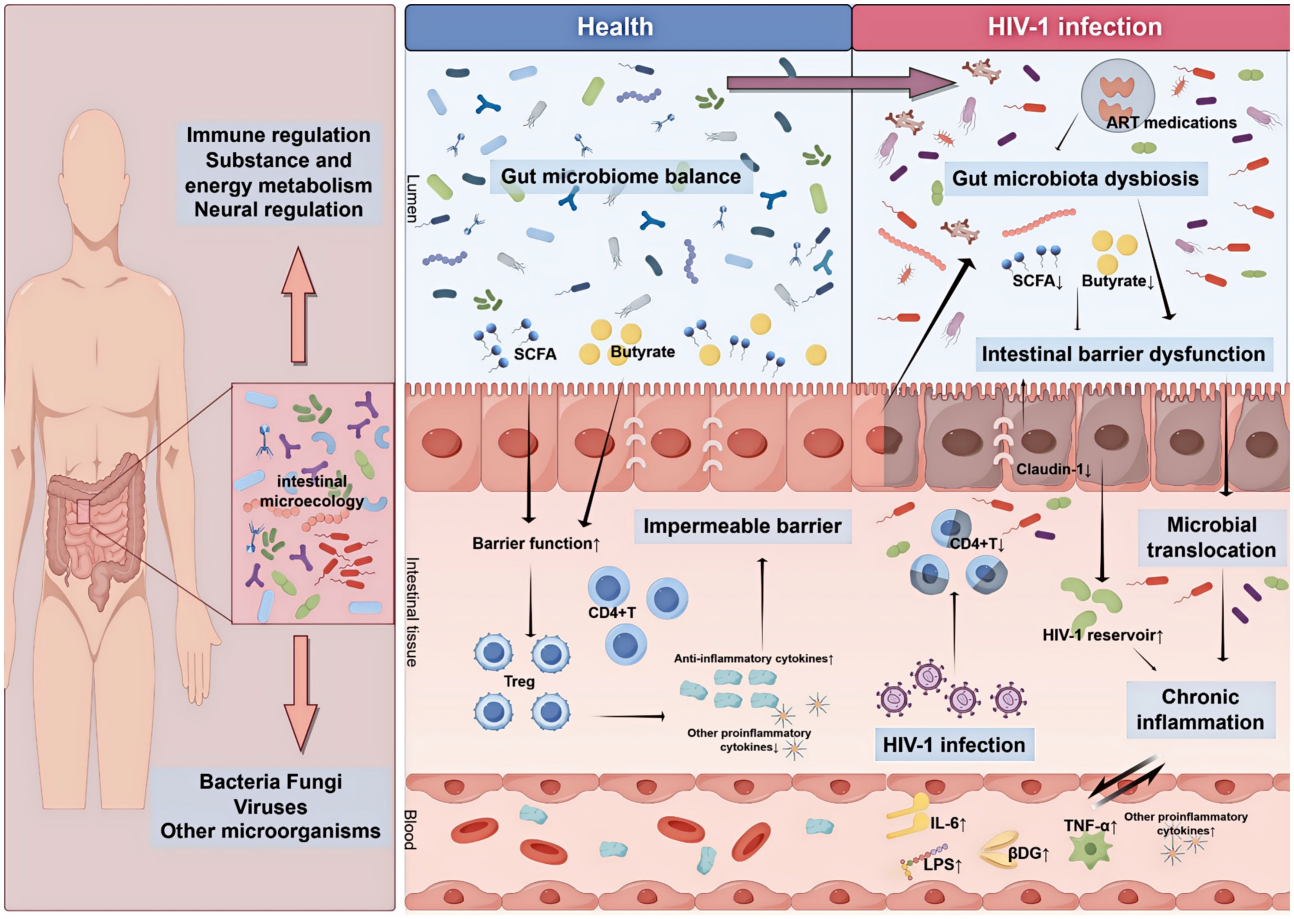
Effect of intestinal microbiota on INR in HIV-1 infection. HIV-1 infection damages CD4 + T cells, leading to gut microbiota dysbiosis and intestinal barrier dysfunction. This results in microbial translocation, triggering persistent chronic inflammation and immune activation in PLWH.

The intestine serves as the primary target organ for HIV-1 replication and reservoir establishment. It contains abundant lymphoid tissue that is essential for maintaining intestinal homeostasis and overall human health. This lymphoid tissue harbors a rich population of intestinal mucosa-associated lymphoid cells, approximately 40% of which are CD4 + T cells. During acute HIV-1 infection, apoptosis of intestinal CD4 + T cells is markedly increased (24, 25). In PLWH, persistent HIV-1 infection causes continuous destruction of these cells, leading to gut microbiota dysbiosis, altered intestinal immunity, and changes in microbiome structure and abundance. As a consequence, the production of short-chain fatty acids (SCFAs), including butyrate, and Claudin-1 is reduced, thereby exacerbating intestinal barrier dysfunction. Subsequently, microbiota and their metabolic products undergo dysregulation and translocate through the compromised mucosal barrier into intestinal tissues and the bloodstream. This process reactivates HIV-1 reservoirs and increases plasma levels of inflammatory mediators, including IL-6, LPS, and TNF-*α*. Consequently, chronic inflammation and persistent immune activation develop, ultimately impairing immune reconstitution following ART (Figure 1). The following sections discuss in detail the mechanisms linking intestinal microecology and INR (Table 1).

**Table 1:** Pathways and research advances related to intestinal microbiota under INR conditions.

Impact pathways	Research progress	References
Intestinal microbiota	Bacteria: (1) Reduced microbial community richness and evenness; (2) Reduced microbial biomass and *α*-diversity; (3) Increased pathogenic symbionts and decreased beneficial microbiota (Proteobacteria↑, Enterococcus↑, Streptococcus↑, Actinobacteria↑, Micrococcaceae↑, Enterobacteriaceae↑).	(26–28)
	Fungi: (1) Altered fungal diversity and composition; (2) Overgrowth of specific fungi (Pseudohyphomyces↑, Bjerkandera↑, *Saccharomyces cerevisiae*↑).	(29, 30)
	Virome: (1) Structural alterations in the intestinal virome and emergence of novel viruses.	(32–34)
ART	(1) ART efficacy correlates with gut microbiota composition (Morganella↑, facultative anaerobes↑, obligate anaerobes↓), driving persistent chronic inflammation; (2) Persistent gut damage post-ART, with incomplete microbiota normalization (Allisonella↑, Lactobacillaceae↓, Peptococcus↓, Collinsella↓); (3) Metabolic processing of ART drugs affects gut microbiota composition (Fusobacteria↑, Proteobacteria↑, Tenella↑, Bacteroidetes↓, Phylum Firmicutes↓).	(35–39)
HIV-1 reservoirs	(1) Correlation between reservoir size and gut microbiome changes affecting immune activation (Prevotella spp.↑, Bacteroides↑, *Burkholderia multivorans*↓, *Bacillus thuringiensis*↓, Clostridioides↓).	(43–45)
Intestinal barrier	(1) Impaired barrier function and increased permeability triggering microbial translocation (Claudins-1↓, CD38↑, HLA-DR↑, caspase-3↑, TNF-α↑); (2) Elevated intestinal-homing CD4 + T cells as key prognostic markers; (3) Reduced production of SCFAs and beneficial gut metabolites (Muribaculaceae↓, Lachnospiraceae↓, Ruminococcaceae↓); (4) Imbalance in Th17/Treg ratio.	(49–58)
Intestinal metabolites	(1) Blood microbiome induces inflammatory responses, impairing immune recovery post-ART (*Porphyromonas gingivalis*↑, Burkholderia multocida↓, *Bacillus thuringiensis*↓); (2) Elevated serum intestinal metabolites and inflammatory biomarkers (IL-6↑, LBP↑, Lactobacillus↑, *Enterococcus durans*↑, L-tryptophan↑, phenylethylamine, Th17↓, IL-17↓, IL-22↓, butyrate-producing bacteria abundance↓); (3) Increased fungal gut colonization (plasma βDG↑).	(43, 55, 59, 63, 64)

### 3.1 HIV-1 infection affect the intestinal microbiota

Following HIV-1 infection, PLWH exhibit characteristic virus-associated alterations in the intestinal microbiota, primarily manifested by reduced microbial biomass and *α*-diversity, increased pathogenic commensals, and depletion of beneficial bacterial populations. Zhou et al. (26) reported that fecal microbiota α-diversity was significantly reduced in PLWH, accompanied by higher abundances of *Proteobacteria*, *Enterococcus*, *Streptococcus*, *Lactobacillus*, *Lachnospiraceae*, *Ruminococcus gnavus*, and *Streptococcus vestibularis*. Lu et al. (27) observed a decreased abundance of *Ruminococcaceae* in INRs with CD4 + T cell counts <200/μL, along with elevated serum pro-inflammatory cytokines, including TNF-*α*, IP-10, and IL-1α. A cross-sectional study further demonstrated significant compositional differences in intestinal flora between immune responders (IRs) and INRs, with INRs showing greater enrichment of pathogenic, opportunistic, and pro-inflammatory bacteria, as well as increased abundance of *Actinobacteria*, *Micrococcaceae*, and *Enterobacteriaceae*. These microorganisms may translocate across the compromised intestinal barrier into the circulation, thereby amplifying systemic inflammation in INRs (28).

HIV-1 infection also induces alterations in intestinal fungal diversity and composition. Meng et al. (29) reported significantly increased abundances of *Pseudohyphomyces*, *Bjerkandera*, and *Saccharomyces cerevisiae* in the intestines of PLWH. Moreover, disruption of the bacterial microbiota and depletion of CD4 + T cells during HIV-1 infection may promote fungal overgrowth and enhance fungal translocation, contributing to chronic inflammation. Accordingly, structural changes in the gut mycobiome may be closely associated with immune recovery following ART (30).

In addition, the human intestine harbors numerous phages as well as eukaryotic RNA and DNA viruses, with phages predominating (31). Although the human intestinal virome remains relatively stable, marked alterations can occur in the intestinal virome of PLWH. Relevant studies have identified multiple novel viruses within the intestinal microecology of PLWH, and these changes in intestinal viral composition are closely associated with AIDS progression. Controlling destructive enteroviral proliferation could potentially reduce gastrointestinal tract damage, thereby limiting immune impairment in PLWH. Thus, alterations in the intestinal virome may negatively impact immune recovery and reconstitution in PLWH (32–34).

### 3.2 ART affect the intestinal microbiota

ART specifically targets retroviruses primarily by inhibiting viral reverse transcriptase activity, thereby effectively blocking viral replication and controlling infection. The intestinal microbiota of PLWH undergoes significant alterations both prior to and following ART initiation. Shi et al. (35) found that PLWH who fail to achieve adequate immune reconstitution after ART exhibit significantly increased intestinal *Morganella* species, accompanied by elevated expression of GT25 enzymes. These findings suggest that the effectiveness of immune reconstitution after ART is closely related to gut microbiota composition, and that such dysbiosis may contribute to impaired immune recovery, potentially serving as a predictive marker. The unique gut microbial changes in PLWH post-ART may also be associated with persistent chronic inflammation. Research by Ishizaka et al. (36) indicated that despite effective ART, PLWH continue to exhibit persistent dysbiosis characterized by reduced obligate anaerobes and increased facultative anaerobes. This microbial imbalance strongly correlates with a pro-inflammatory immune state, potentially driving chronic inflammation and related complications.

Long-term ART does not fully reverse intestinal microbiota disruption caused by HIV-1 infection. Ancona et al. (37) reported a reduced abundance of *Lactobacillaceae*, *Peptococcus*, and *Collinsella*, along with the presence of *Allisonella* or *Desulfovibrio* following ART treatment. They also suggested a potential association between *Veillonellaceae* and intestinal fatty acid-binding protein (I-FABP) levels, indicating persistent intestinal damage. Although ART effectively inhibits HIV-1 replication, intestinal microbiota composition does not completely return to normal. Furthermore, the metabolic processes involved in ART drugs may influence intestinal microbiota composition and function (38, 39). One study evaluating the impact of ART on intestinal microbiota composition found that ART initiation was associated with increased abundances of *Fusobacteria* and *Proteobacteria*, a relative increase in Prevotella, and decreased abundances of *Bacteroidetes* and *Firmicutes*, indicating a reduction in intestinal microbiota diversity following ART (40).

### 3.3 Intestinal microbiota dysregulation affects HIV-1 reservoirs

The HIV-1 viral reservoir refers to persistent infection with replication-competent HIV-1 in patients receiving long-term effective ART, primarily composed of resting memory CD4 + T cells (41). HIV-1 DNA, an essential intermediate product in viral replication, constitutes the main component of the HIV-1 reservoir. It reflects the overall reservoir size in PLWH, with viral load levels correlating with disease progression and treatment efficacy. Early initiation of ART during acute infection decreases total HIV-1 DNA and RNA levels, thus limiting the size of the viral reservoir. However, the stable population of latently infected CD4 + T cells within the reservoir is not significantly reduced by early ART (42).

Characteristics of the intestinal microbiome correlate with HIV-1 reservoir size and may influence immune reconstitution in PLWH. Guo et al. (43) identified blood-enriched microbial communities, such as *Prevotella* spp., originating from intestinal translocation. These bacteria positively correlated with HIV DNA and RNA levels, suggesting their potential role in maintaining the viral reservoir. Certain bacteria abundant in healthy controls (e.g., *Burkholderia multivorans*, *Bacillus thuringiensis*) were significantly reduced in PLWH. Their abundance positively correlated with CD4 + T cell counts and negatively correlated with HIV DNA/RNA levels, indicating that their absence may impair immune reconstitution and stabilize the viral reservoir. Borgognone et al. (44) observed that compared with non-controllers, viral load controllers exhibited a higher *Bacteroides/Clostridioides* ratio and lower microbial gene richness. The *Bacteroides*/*Clostridioides* ratio negatively correlated with viral reservoir size (HIV-1 DNA and cell-associated HIV-1 RNA). Individuals with a high *Bacteroidetes/Clostridiales* ratio demonstrated gene expression patterns associated with immune activation. Nonetheless, further studies are required to clarify interactions among the gut microbiome, immune system, and HIV-1 reservoir (45).

### 3.4 HIV-1 infection disrupts the intestinal barrier

The intricate tight junctions between intestinal mucosal cells are critical for preserving mucosal integrity, and Claudin-1, a core component of these junctions, significantly influences intestinal epithelial permeability (46–48). HIV-1 infection induces marked apoptosis of intestinal CD4 + T cells, damaging the intestinal mucosal barrier and increasing permeability. This damage results in microbial translocation, commonly referred to as “leaky gut,” wherein microbes (bacteria and fungi) and their products enter systemic circulation (49). This phenomenon is closely associated with chronic inflammation and persistent immune activation, characterized by elevated expression of CD38 and HLA-DR, both key factors negatively affecting immune reconstitution following ART (50). Guo et al. (51) reported a significant reduction in Claudin-1 expression and increased caspase-3 and TNF-*α* expression levels in intestinal epithelial tissues of INRs. These findings suggest enhanced epithelial permeability and increased tissue damage, including glandular apoptosis, accompanied by systemic inflammatory responses. Such intestinal epithelial damage may persist in PLWH despite long-term ART.

The increased levels of gut-homing CD4 + T cells, resulting from intestinal barrier impairment and microbial translocation, may similarly hinder immune reconstitution in PLWH receiving ART (52). Lorvik et al. (53) found that INRs exhibited higher numbers of gut-homing CD4^+^ T cells in peripheral blood compared with IRs. In contrast, the fraction of gut-homing CD4 + T cells was negatively correlated with the CD4: CD8 ratioo, indicating that elevated gut-homing cells might serve as an important prognostic marker for poor outcomes in PLWH undergoing ART. Similarly, Rousseau et al. (54) demonstrated that PLWH receiving long-term ART had incomplete peripheral CD4 + T-cell recovery, characterized by activated CD8 + T cells and increased intestinal homing of CD4 + T cells.

SCFAs are important mediators of intestinal immune responses and maintenance of intestinal epithelial integrity (55). Studies have reported significantly decreased abundances of *Muribaculaceae*, *Lachnospiraceae*, and *Ruminalococcaceae* in the intestines of PLWH. Such alterations may reduce the production of SCFAs and other beneficial intestinal metabolites, further compromising intestinal barrier integrity and exacerbating microbial translocation (56). Additionally, intestinal CD4 + T cells, particularly helper T cells of the Th17 subtype, are crucial for mucosal immune responses. Since Th17 cells express mucosal homing receptors (CCR6/α4β7) and HIV-1 receptors and co-receptors (CD4/α4β7/CCR5/CXCR4), they are highly susceptible to HIV-1 infection and depletion, thereby compromising intestinal barrier integrity (57). Studies have demonstrated that an imbalance in the Th17/Treg ratio closely associates with damage to the intestinal mucosal barrier, which is a characteristic feature in the development of INR after ART (58).

### 3.5 HIV-1 infection alters intestinal metabolites

Intestinal microbiota-derived metabolites serve as critical mediators connecting intestinal microbiota with host functions. These metabolites, generated via microbial biotransformation of exogenous and endogenous compounds, play essential roles in maintaining immune homeostasis, host health, and nutritional status. HIV-1 infection significantly disrupts intestinal microecological equilibrium, profoundly altering microbial metabolic activity due to viral infection and structural and functional intestinal changes, consequently leading to adverse outcomes. Guo et al. (43), employing metagenomic sequencing and Olink proteomics, analyzed microbial profiles and inflammation-related proteins in peripheral blood samples. Their findings revealed persistent microbial translocation in peripheral blood of INRs undergoing ART, characterized by a significant increase in *Porphyromonas gingivalis* and significant decreases in *Burkholderia multocida* and *Bacillus thuringiensis*. These changes correlated positively with inflammation-associated proteins, indicating that blood microbiota may influence immune recovery post-ART by eliciting inflammatory responses and altering inflammation-related protein profiles. In a multicenter cohort study involving men who have sex with men (MSM), Chen et al. (59) observed pathogenic alterations in intestinal microbiota, accompanied by significantly elevated plasma levels of inflammatory cytokines (IL-6 and lipopolysaccharide-binding protein [LBP]). These findings suggest that increased intestinal metabolites and inflammatory biomarkers in blood may correlate with heightened AIDS risk and affect ART efficacy.

Butyrate plays a pivotal role in reshaping the intestinal microenvironment (60), facilitating mucus production and strengthening tight junction-mediated intestinal barrier integrity. Reduced butyrate production may exacerbate inflammatory responses and mucosal HIV-1 replication (61, 62). Dillon et al. (55) found a significant reduction in intestinal abundance of butyrate-producing bacteria (primarily *Roseburia intestinalis*) in PLWH. This decline in butyrate-producing bacteria leads to decreased butyrate levels, further damaging the intestinal epithelial barrier and exacerbating microbial translocation. Even after ART, some patients may exhibit persistent reductions in the relative abundance of butyrate-producing bacteria.

In a case–control study, Zhang et al. (63) identified elevated intestinal abundances of *Lactobacillus* and *Enterococcus durans* in PLWH, resulting in increased concentrations of L-tryptophan and phenylethylamine. L-tryptophan, metabolized by indoleamine 2,3-dioxygenase 1 (IDO1), generates tryptophan metabolites that inhibit differentiation of Th17 cells and reduce production of IL-17 and IL-22 by natural killer (NK) cells, thus promoting microbial translocation. Simultaneously, high concentrations of phenylethylamine trigger shedding of intestinal epithelial cells, closely associated with microbial translocation and immune activation. Collectively, these findings underscore that intestinal metabolites significantly influence immune reconstitution by acting upon the intestinal barrier.

Studies have observed increased intestinal fungal colonization and elevated plasma βDG in PLWH during HIV-1 infection, persisting even after long-term ART. Similar to LPS, βDG contributes to chronic immune activation and INR events in PLWH (48). Ramendra et al. (64) assessed the relationship between LPS and βDG as biomarkers for ART progression and immune activation, highlighting βDG as an emerging source of monocyte and NK cell activation leading to immune dysfunction. Elevated plasma βDG levels may reflect systemic immune activation, emphasizing the significant role of fungal translocation.

## 4 Other factors affecting the intestinal microecology in PLWH

Beyond the aforementioned factors, coinfections, nutritional status, and socioeconomic conditions may also influence the interaction between intestinal microecology and immune function in PLWH, thereby impairing immune reconstitution following ART.

### 4.1 Coinfections

Coinfections negatively affect immune recovery in PLWH after ART by inducing excessive immune activation, exacerbating intestinal barrier damage, and disrupting gut microbiota. For example, Negash et al. (65) investigated the impact of tuberculosis (TB) coinfection on immune reconstitution after ART in PLWH. Their results showed that patients with concurrent TB coinfection experienced a higher incidence of immune reconstitution failure, indicating a substantial negative effect of coinfections on immune recovery. Besides TB, impaired immune function during ART frequently facilitates coinfections with other viruses, including hepatitis B virus (HBV), hepatitis C virus (HCV), cytomegalovirus (CMV), and Epstein–Barr virus (EBV). These viral coinfections can hinder CD4 + T-cell recovery and immune reconstitution. Studies have specifically demonstrated that CMV or EBV coinfections in PLWH elevate plasma inflammatory cytokine levels, thereby promoting chronic immune activation. Moreover, ongoing CMV and EBV replication significantly increases HIV-1 DNA diversity, potentially contributing to chronic HIV-1 persistence (66, 67).

### 4.2 Nutritional status

Regarding patient nutritional status, Seid et al. (68) demonstrated that early detection and intervention for malnutrition in PLWH support immune system recovery and delay HIV-1 disease progression. Damage to intestinal barrier integrity severely impairs nutrient absorption, further hindering immune recovery in PLWH. Studies (69, 70) have reported that PLWH receiving long-term ART often experience varying degrees of malnutrition. Systematic reviews and meta-analyses (68, 71) indicated that CD4 + T-cell counts below 350 or 200 cells/μL correlate significantly with malnutrition, identifying malnutrition as a critical risk factor for INR. Throughout HIV-1 infection, the patient’s nutritional status is closely linked to immune function. Malnutrition-induced alterations in immune cell populations, hormones, and cytokine profiles disrupt immune cell metabolism, leading to immunosuppression and possibly contributing to INR pathogenesis after ART (68, 72). Additionally, dietary patterns and nutritional intake levels significantly influence gut microbiota composition and function. High-fiber diets promote beneficial bacterial growth, whereas diets high in fat and sugar may induce microbiota dysbiosis. Manzano et al. (73) evaluated dietary patterns and their effects on gut microbiota and inflammatory markers in PLWH, concluding that a Mediterranean-style diet enhances gut microbial structure, reduces inflammation, and benefits long-term health. These findings suggest that nutritional interventions may alleviate chronic inflammation and support immune recovery.

### 4.3 Socioeconomic conditions

Socioeconomic factors, including educational level, healthcare accessibility, and living conditions, influence how intestinal microecology affects INR. Poor socioeconomic status is associated with inadequate sanitation and limited healthcare resources, increasing infection risk and impairing nutritional status, thereby hindering immune reconstitution. To fully elucidate the mechanisms linking intestinal microbiota and INR, it is essential to rigorously control for these socioeconomic confounders.

## 5 Microbiota-based interventions

Alterations in intestinal microbiota during HIV-1 infection are closely associated with immune dysfunction. Importantly, the microbiota represents a key factor influencing INR in PLWH receiving ART by driving local and systemic inflammation, immune activation, and immune dysregulation. Consequently, precision interventions aimed at restoring microbiota balance may facilitate immune reconstitution by reducing inflammation and immune dysregulation, ultimately improving INR outcomes. With advancements in research, developing and implementing effective personalized interventions and standardized clinical protocols is imperative to improve INR management. The following discussion highlights opportunities for microbiota-based interventions (Table 2).

**Table 2:** Intestinal microbiota-based interventions.

Treatment methods	Research progress		References
FMT	(1) Restores gut microbiome composition and enhances microbial diversity in PLWH. (2) Reduces recurrent infection risk. (3) Lowers inflammatory protein levels. (4) Promotes colonization by beneficial butyrate-producing bacteria. (5) Improves intestinal barrier integrity.		(75–79)
Probiotics	(1) Increases CD4 + T cell counts and alleviates HIV-1-associated symptoms. (2) Decreases sCD14 levels and microbial translocation. (3) Reduces intestinal cell apoptosis and restores barrier integrity. (4) Enhances abundance of beneficial microbiota.		(81–85)
TCM	Active ingredients	(1) Artemisinin: Reduces early CD4 + T-cell apoptosis and increases probiotic abundance. (2) TPL: Inhibits IFN signaling pathway and STAT1 phosphorylation, improving immune reconstitution.	(90, 91)
	Single TCM	(1) TwHF: Increases CD4 + T-cell counts and suppresses immune activation. (2) Probiotic-fermented herbs: Restores gut microbiota and repairs intestinal barrier.	(91, 93)
	Compound decoctions	(1) Yi’ai Kang Capsules: Reduce HIV-1 viral replication, mitigate immune damage, and restore intestinal microbiota balance.	(94)

### 5.1 Fecal microbiota transplantation

Fecal microbiota transplantation (FMT) involves transferring fecal matter from healthy donors to patients whose conditions are linked to intestinal microbiota dysbiosis, aiming to restore microbial balance and achieve therapeutic benefits (74). Several studies have investigated whether FMT can effectively restore the gut microbiome in PLWH. For instance, a systematic review examining FMT for Clostridioides difficile infections in immunocompromised patients demonstrated that FMT helped restore typical microbial communities in patients both with and without *C. difficile* infection, reducing recurrent infection risks in PLWH receiving ART (75, 76). Further building upon these findings, a randomized, single-blind, two-group pilot study by Routy et al. (NCT06022406) investigated whether FMT could reduce mucosal and systemic inflammation in PLWH receiving ART with low CD4/CD8 ratios. This study also examined potential impacts on HIV-1 reservoirs, which could support enhanced long-term health management (77).

Díaz-García et al. (78) evaluated the effects of weekly FMT versus placebo treatment over 8 weeks on systemic inflammatory proteomic profiles in 29 PLWH receiving ART. Results demonstrated that FMT significantly reduced plasma levels of 45 inflammatory proteins, indicating that targeting gut microbiota through FMT effectively decreases systemic inflammation in PLWH, with sustained effects. Serrano-Villar et al. (79) reported that oral encapsulated FMT was safe, well-tolerated, and effectively alleviated HIV-1-associated gut dysbiosis in PLWH receiving ART. This intervention significantly enhanced microbial diversity and promoted colonization by beneficial butyrate-producing bacteria. Simultaneously, FMT markedly improved the intestinal barrier damage marker intestinal fatty acid-binding protein (IFABP), offering a novel therapeutic strategy for mitigating chronic HIV-1-associated inflammation through microbiota modulation. However, as an emerging therapeutic approach in HIV treatment, further comprehensive studies are required to elucidate the precise mechanisms through which FMT modulates intestinal microbiota composition and influences inflammatory and immune pathways in PLWH.

### 5.2 Probiotics

Probiotics significantly influence host health by maintaining intestinal microbiota balance and exhibit notable therapeutic efficacy for treating specific diseases (80). Supplementation with probiotics is expected to increase CD4 + T cell counts, alleviate HIV-1-associated symptoms, and concurrently restore gut microbiota equilibrium and immune function in PLWH (81). Supporting this, Blázquez-Bondia et al. (82) evaluated the efficacy and safety of long-term probiotic supplementation in PLWH experiencing INR after prolonged ART. Their findings demonstrated that a synbiotic intervention combining *Lactobacillus plantarum* and Pseudomonas acidophilus strains with prebiotic fiber was safe, increased the CD4+/CD8 + ratio, modestly reduced sCD14 levels, and effectively diminished intestinal microbial translocation.

Probiotics were also closely associated with reduced T cell activation, restored intestinal epithelial barrier integrity, decreased intraepithelial lymphocyte density, and reduced intestinal cell apoptosis. These beneficial effects coincided with increased abundances of Faecalibacterium and Prevotella genera, enhanced butyrate production, and improved inflammatory biomarker levels, including soluble CD14 and high-sensitivity C-reactive protein (83). In addition, probiotics promoted increased abundances of active bacteria with potent immunomodulatory properties involved in amino acid metabolism, aligning microbial profiles more closely with those observed in healthy individuals (84, 85). Future research should further explore optimal probiotic and prebiotic ratios and combinations, clearly define probiotic species selection, intervention duration, and establish standardized guidelines for their clinical use.

### 5.3 Traditional Chinese medicine

TCM employs plant-based botanicals for their medicinal properties and therapeutic effectiveness against infectious diseases (86, 87). Due to its multicomponent, multitarget, multipathway, and bidirectional regulatory characteristics, TCM acts after gastrointestinal absorption to influence the intestinal microbiota. It enhances microbiota composition, repairs the mucosal barrier, and regulates intestinal metabolites. These actions collectively boost immune function and expand therapeutic strategies for managing INR (88, 89).

Artemisinin, a sesquiterpene derivative with antiviral and immunomodulatory effects, has been explored in HIV-1 research and treatment. Clinical studies demonstrated that after 48 weeks of oral artemisinin administration, PLWH exhibited improved baseline T-cell counts, reduced early apoptosis of CD4 + T cells, elevated circulating CD4 + T-cell levels, and accelerated immune reconstitution in INR (2, 90). In addition to artemisinin, Tripterygium wilfordii Hook F (TwHF), a traditional Chinese herb used to treat autoimmune and inflammatory disorders, has demonstrated clinical efficacy in managing INR. In a study involving 33 INR patients, a median treatment duration of 17 months with TwHF significantly increased CD4 + T-cell counts while reducing expression of T-cell activation markers, suggesting that TwHF promotes immune reconstitution and suppresses immune activation. To elucidate TwHF’s pharmacological mechanism, researchers combined clinical multi-omics analysis, network pharmacology, and *in vitro* validation, revealing that triptolide (TPL), the primary bioactive compound in TwHF, inhibits IFN signaling and phosphorylation of the critical molecule STAT1, thus improving immune reconstitution in INR (91).

Herbal medicines fermented with probiotics can transform herbal components into novel bioactive molecules and enhance the release of active constituents, such as organic acids and polysaccharides, thereby providing new pharmacological properties (92). Studies indicate that probiotic-fermented herbs positively modulate the intestinal microbiota and barrier integrity, exerting synergistic effects on intestinal microecology and facilitating immune recovery (93).

Compound decoctions, the most common form of herbal preparation, are frequently used in INR treatment. For example, Yi’ai Kang Capsules, a complex herbal formulation containing ginseng, astragalus, white peony root, fried white atractylodes, *Rehmannia glutinosa*, and *Ophiopogon japonicus*, possess multiple pharmacological properties, including dispelling wind and heat and resolving dampness. Yi’ai Kang Capsules can restore Th17/Treg cell balance, effectively ameliorate gut microbiota dysbiosis, enhance immune function, and simultaneously reduce viral replication and immune injury by modulating the NF-κB signaling pathway (94). Based on these observations, future TCM research should incorporate multi-omics technologies to comprehensively investigate its therapeutic pathways and underlying mechanisms.

Moreover, dietary modifications and oral vitamin D supplementation represent important approaches to modulate intestinal microbiota. Currently, specific dietary interventions targeting INR are lacking, and investigations on vitamin D supplementation among PLWH receiving ART remain limited. Further research is necessary to evaluate their potential regulatory effects on INR (73, 95).

## 6 Summary and future perspectives

The intestinal microbiome, a complex microbial ecosystem closely linked to human health, has increasingly been recognized as a critical factor influencing immune reconstitution in PLWH receiving ART. Modulating intestinal microecology may represent an innovative therapeutic strategy for treating INR. This review systematically elucidates mechanisms linking intestinal dysbiosis (including bacteria, fungi, and viruses), intestinal barrier damage, and abnormal microbial metabolite profiles to INR. These disturbances exacerbate microbial translocation, induce chronic immune activation and inflammation, and contribute to immune exhaustion, ultimately hindering CD4 + T-cell recovery. Interventions targeting intestinal microbiota, such as fecal microbiota transplantation, probiotics, and specific TCM formulations, show significant potential to restore microbial homeostasis and improve immune function. These approaches have been validated through preclinical research and preliminary clinical trials.

However, research in this area remains at an early stage, characterized by methodological limitations. The human intestinal microbiome is influenced by numerous factors, including diet, physical activity, medication use, and psychological status. Most studies present fragmented findings, focusing independently on gut microbes or intestinal barrier function. The absence of large-scale, evidence-based investigations limits the translation of research into effective INR treatments. Future research should prioritize conducting large-scale, multicenter clinical trials to validate microbiome interventions. Immunological assays, genomic sequencing, and transcriptomics should be employed to elucidate the mechanisms of immune exhaustion associated with microbiome imbalance. These efforts may uncover novel therapeutic targets and reliable biomarkers for predicting and monitoring INR and treatment responses. Ultimately, precise modulation of intestinal microbiota will facilitate the development of personalized diagnostic and therapeutic strategies, significantly improving long-term outcomes for PLWH.
